# Retail Frozen Blended Beverages as Vehicles for Opportunistic Antibiotic‐Resistant and Biofilm‐Forming Bacteria

**DOI:** 10.1155/ijm/3043052

**Published:** 2026-10-23

**Authors:** Qutaiba Ababneh, Ekhlas Al-Rousan, Ziad Jaradat, Ghazal Almomani, Jana Al-Obaidat, Rula Alazzeh, Shahed Ogiliat, Farah Al-Najjar

**Affiliations:** ^1^ Department of Biotechnology and Genetic Engineering Jordan University of Science and Technology Irbid Jordan just.edu.jo

**Keywords:** antimicrobial resistance, biofilm formation, cross-contamination, milkshake, slushy drinks, smoothie

## Abstract

The consumption of milkshakes, slushies, and smoothies has increased due to their refreshing taste and nutritional benefits. However, the microbiological quality of these beverages poses significant public health concerns. This study is aimed at investigating the prevalence of pathogenic bacteria in these popular beverages, addressing a critical gap in food safety research. A total of 150 samples of milkshakes, smoothies, and slushies were collected, and the target bacteria were identified using microbiological and molecular methods. The obtained results revealed the prevalence of *coliforms* (50%), *Klebsiella* spp. (37.3%), *Escherichia coli* (9.3%), *Acinetobacter* spp. (4.6%), *Listeria* spp. (4.6%), and *Staphylococcus* spp. (4%) in the collected samples. Most isolates were sensitive to the tested antibiotics. *Acinetobacter baumannii* isolates exhibited high resistance levels against tetracycline (71.4%) and doxycycline (57.1%). The highest resistance among the 14 *E*. *coli* isolates was observed against ampicillin and sulfonamides (78.6%), while highest resistance rate among the *Klebsiella* isolates was observed against ampicillin (71.4%). Biofilm‐forming ability varied among the isolates. Most *Staphylococcus* isolates (66.7%) and *Acinetobacter* isolates (71.4%) demonstrated strong biofilm‐forming capacity, whereas the majority of *Listeria* isolates (85.7%) showed weak biofilm formation. These findings underscore the need for quality control measures to ensure continuous monitoring and enhancement of microbial safety of these products.

## 1. Introduction

Smoothies, milkshakes, and slushies are widely consumed beverages throughout the year, especially in summer. Milkshake drinks are prepared by blending milk and ice cream with fruits and served with cream as a topping [1]. Milk is the main component of milkshake drinks and is considered a potential source of contamination by pathogenic bacteria such as *Acinetobacter baumannii* [2]. Smoothie drinks are prepared by blending different raw fruits and vegetables [3]. Although smoothies, milkshakes, and slushies are considered healthy drinks due to the use of fresh produce and dairy products, they are vulnerable to microbial contamination due to the lack of heat treatment or thorough cleaning as in the case of fruits and vegetables [3].

Microbial contamination of smoothies, milkshakes, and slushies could occur from several sources. Fresh fruits and vegetables have high levels of natural sugars and water activity, making them highly prone to the growth of pathogenic microorganisms [4]. Moreover, the water used in the preparation of drinks or the washing of machines can be contaminated by microorganisms such as coliform bacteria [5]. Additionally, fresh fruits and vegetables can be contaminated by different types of microorganisms during their cultivation, harvesting, postharvest handling, processing, and distribution, and through fertilization or watering processes [6–8]. The Center for Disease Control and Prevention (CDC) reported that contaminated fruits and vegetables are the cause of almost 46% of foodborne illnesses in the US through the period 1998–2008 [9]. Lastly, the dairy products used in the preparation of milkshakes are highly prone to the growth of several pathogenic bacteria owing to their high content of nutrients [10].

Microbial contamination of drinks is a critical concern due to its potential impact on human health, as microbial agents can cause fatal foodborne illnesses. Symptoms of diseases associated with ingesting contaminated food can vary from mild gastrointestinal issues to critical conditions affecting the nervous system, liver, or kidneys. Members of the *Clostridium*, *Bacillus*, *Listeria*, *Salmonella*, *Staphylococcus*, *Campylobacter*, and *Vibrio* genera and some strains of *Escherichia coli* were linked to cause more than 90% of foodborne diseases [11]. Several studies have reported the isolation of different pathogenic and nonpathogenic bacteria from smoothies, including *Acinetobacter*, *E*. *coli*, *Klebsiella*, *Enterobacter*, *Citrobacter*, *Enterococcus*, *Staphylococcus*, *Pseudomonas aeruginosa*, *Bacillus*, *Cronobacter sakazakii*, and *Salmonella* [3, 4, 12–15]. Milkshakes have been reported to be contaminated with *E*. *coli*, *Staphylococcus aureus*, *Enterococcus*, *Klebsiella*, *Proteus*, *Pseudomonas*, *Bacillus*, *Micrococcus*, *Streptococcus*, *Shigella*, *Aeromonas*, *Micrococcus*, *Citrobacter*, *Salmonella*, and *Listeria* [1, 5, 10, 16–18].

Recent studies have documented the increasing prevalence of antimicrobial‐resistant foodborne pathogens associated with dairy and other food products. The widespread and inappropriate use of antibiotics in food‐producing animals has facilitated the emergence of resistant bacterial strains, representing a significant public health concern. Furthermore, biofilm formation by food‐associated microorganisms has received considerable attention because biofilms enhance bacterial survival, persistence, and resistance to disinfectants and antimicrobial agents. Biofilms that develop on food‐processing surfaces can act as persistent sources of contamination, thereby compromising both food safety and quality. Consequently, elucidating the relationship between antimicrobial resistance and biofilm formation is critical for improving food hygiene and developing effective control strategies [19–24].

Given their global consumption, the microbial safety of smoothies, milkshakes, and slushies is a universal public health concern. However, food safety risks are often localized, and the complete lack of data from Jordan represents a knowledge gap. Therefore, this study is aimed at establishing the prevalence of bacterial contamination in these beverages sold in Jordan. Additionally, the study assessed the broader public health threat by identifying key pathogens and characterizing their antibiotic resistance and biofilm‐forming capabilities.

## 2. Materials and Methods

### 2.1. Samples Collection and Isolation of Different Types of Bacteria

Between May and October 2023, a total of 150 samples of milkshakes (*n* = 41), smoothies (*n* = 87), and slushies (*n* = 22) were collected from different local shops in Irbid, Jordan. Samples were randomly collected by selecting four different sample types from each shop, transporting them to the laboratory in an ice box, and processing them immediately. The isolation process of all bacteria started by adding 25 g of each sample to 225 mL of buffered peptone water (BioMaxima, Poland). The samples were incubated overnight at 37°C. After that, 10 mL of each sample was added to 90 mL of Enterobacteriaceae Enrichment Broth‐Mossel (BioMaxima, Poland) and incubated overnight at 37°C [25]. On the same day, each sample was streaked on CHROMagar *Acinetobacter* agar (CHROMagar, France), cetrimide agar (BioMaxima, Poland), mannitol salt agar (Biolab, Hungary), *Listeria* selective agar (Oxoid, United Kingdom), and violet red bile agar (Oxoid, United Kingdom) and incubated at 35°C for 1–3 days depending on the type of bacteria to isolate *Acinetobacter*, *P*. *aeruginosa, Staphylococcus*, *Listeria*, and *coliforms*, respectively. After the second overnight incubation, all samples were streaked on MacConkey agar (Condalab, Spain), eosin methylene blue agar (Condalab, Spain), Brilliance *Enterobacter Sakazakii* Agar (Oxoid, United Kingdom), violet red bile agar (Oxoid, United Kingdom), and *Salmonella Shigella* agar (Condalab, Spain) and incubated at 35°C for 1–3 days depending on the type of bacteria to isolate *Klebsiella*, *E*. *coli*, *C*. *sakazakii*, *coliforms*, *Salmonella*, and *Shigella*, respectively. Presumptive colonies of all bacterial types were preserved at −80°C for further analyses. All procedures were carried out under aseptic conditions using a laminar flow hood.

### 2.2. Total Bacterial Count (TBC) and Total Coliform Count (TCC)

TBC and TCC were determined for the pre‐enrichment and postenrichment samples. For the pre‐enrichment samples, 100 *μ*L was taken from each sample and added to 900 *μ*L of buffered peptone water, serially diluted from 10^−1^ to 10^−6^, and plated from each dilution and the sample stock on Luria–Bertani (LB) agar (Biolab, Hungary) and violet red bile agar (Oxoid, United Kingdom). For the postenrichment samples, 100 *μ*L was taken from each sample and added to 900 *μ*L of Enterobacteriaceae Enrichment Broth‐Mossel, serially diluted from 10^−1^ to 10^−6^, and plated from each dilution and the sample stock on LB agar (Biolab, Hungary) and violet red bile agar (Oxoid, United Kingdom). After incubating the agar plates overnight, colonies of all dilutions and the stock were counted to calculate the CFU for each sample.

### 2.3. Molecular Identification of Bacteria

The DNA of the presumptive isolates was extracted by the in‐house phenol‐chloroform method. Molecular identification of bacteria was carried out by partial PCR amplification of the *uidA* gene of *E*. *coli* [26], the *gyrA* gene of *Klebsiella* spp. [27], the *phoE* gene of *Klebsiella pneumoniae* [28], the *pehX* gene of *Klebsiella oxytoca* [29], the *lacZ* gene of *coliforms* [30], the *invA* gene of *Salmonella* [31], the *tuf* gene of *Staphylococcus* spp. [32], the *nuc* gene of *S*. *aureus* [33], the *mecA* gene of MRSA [34], the *oprI* gene of *Pseudomonas* spp. [35], and the *oprL* gene of *P*. *aeruginosa* [35]. Moreover, multiplex PCR assays were performed to identify and differentiate between *Acinetobacter* spp., *Listeria* spp., and *Cronobacter* spp., as described previously by Chen et al. [36], Tao et al. [37], and Wang et al. [38], respectively. *Shigella* species were identified by amplifying the *gyrB* gene [39]. Then, PCR products were purified from agarose gels and subjected to dideoxy chain termination sequencing (Macrogen, South Korea). BLAST search was used to analyze the obtained DNA sequences. Presumptive *Shigella* DNA sequences were deposited into GenBank under the Accession Numbers PZ126957‐ PZ127014. More information about these sequences can be found in the Supporting Information (Table S1).

### 2.4. Antibiotic Sensitivity Testing

The Kirby–Bauer disk diffusion method was used to carry out the antibiotic sensitivity testing for all recovered isolates against the antibiotics described in the Clinical and Laboratory Standards Institute (CLSI) guidelines [40] for each bacterium. Three to five fresh colonies from each isolate were suspended in 1 mL of 0.9% (*w*/*v*) normal saline, and the culture turbidity was adjusted to 0.5 McFarland standard (optical density [OD] 0.08–0.13) at 625 nm. A sterile swab was dipped into the suspension and used to streak Mueller–Hinton agar (MHA) (Biolab, Hungary) plates. The desired antibiotic disks were placed on the MHA plates and incubated at 37°C for the time specific for each bacterium [41]. The next day, the diameters of inhibition zones were measured, and the isolates were categorized as susceptible, intermediate, or resistant following the CLSI guidelines [40]. The multidrug‐resistant (MDR) strains were defined as acquired resistance to at least one agent in three or more antimicrobial categories [42].

### 2.5. Biofilm Formation Assay

The ability of the recovered *A*. *baumannii* and *Pseudomonas* isolates to form biofilm was performed as described by Hu et al. [43, 41]. Briefly, one overnight colony for each isolate was inoculated in 3 mL of LB broth (Biolab, Hungary), and then the OD was adjusted to 0.1 at 600 nm. For each isolate, 200 *μ*L were taken from the adjusted culture and used to fill three wells in a 96‐well microtiter plate. The LB broth was used to fill three wells as a negative control. The microtiter plate was incubated at 37°C for 24 h. After incubation, every well was washed gently three times with phosphate‐buffered saline (PBS) to remove unbound bacterial cells, air‐dried and stained with 0.1% crystal violet solution (*w*/*v*) for 20 min. After that, the wells were washed with water four times, air‐dried, and crystal violet was solubilized with 95% ethanol (*v*/*v*), and the plates were scanned at 570 nm to determine the OD of the stained cells. The ODs of 3 independent plates were compared with the cut‐off optical density (ODc) to determine the biofilm phenotype as follows: nonbiofilm producer: OD ≤ ODc; weak biofilm producer: ODc < OD ≤ 2 × ODc; moderate biofilm producer: 2 × ODc < OD ≤ 4 × ODc; or strong biofilm producer: OD > 4 × ODc. *A*. *baumannii* ATCC 19606 and *P*. *aeruginosa* ATCC 9027 were used as positive controls.

The ability of *Klebsiella* isolates to form biofilm was examined by following the same procedure used for *A. baumannii* and *Pseudomonas*, except that the concentration of crystal violet solution was 1% (*w*/*v*). For *E*. *coli*, *Cronobacter*, and *Shigella*, the biofilm formation ability was assayed by following the same procedure with some modification. Briefly, one overnight colony for each isolate was inoculated in 3 mL of supplemented LB broth (Biolab, Hungary) with 2% (*w*/*v*) sucrose, and then the OD was adjusted to 0.1 at 600 nm. For each isolate, 200 *μ*L were taken from the adjusted culture and used to fill three wells in a 96‐well microtiter plate. The LB broth was used to fill three wells as a negative control. The microtiter plate was incubated at 35°C for 24 h. After incubation, every well was washed gently three times with PBS to remove unbound bacterial cells, air‐dried and stained with 1% crystal violet solution (*w*/*v*) for 20 min. After that, the wells were washed with water four times, air‐dried, and crystal violet was solubilized with 95% ethanol (*v*/*v*), and the plates were scanned at 570 nm to determine the OD of the stained cells. The ODs of three independent plates were compared with the ODc to determine the biofilm phenotype as follows: nonbiofilm producer: OD ≤ ODc; weak biofilm producer: ODc < OD ≤ 2 × ODc; moderate biofilm producer: 2 × ODc < OD ≤ 4 × ODc; or strong biofilm producer: OD > 4 × ODc.

For *Staphylococcus* and *Listeria*, the biofilm assay was examined by following the protocol published by Ababneh et al. [44] with some modifications. Briefly, three to five colonies for each isolate were suspended in 1 mL of brain heart infusion (BHI) broth supplemented with 1% (*w*/*v*) glucose, and the culture turbidity was adjusted to 0.5 McFarland standard (OD 0.08–0.13) at 600 nm. For each isolate, 10 *μ*L were taken from the adjusted culture and added to a well filled with 190 *μ*L of BHI broth. The negative control wells contained 200 *μ*L of BHI broth. The microtiter plate was incubated at 37°C for 24 h. After incubation, every well was washed gently three times with 200 *μ*L of PBS to remove unbound bacterial cells, heat fixated at 60°C for 20 min, and stained with 175 *μ*L of 0.5% (*w*/*v*) crystal violet for 5 min. After that, the wells were washed four times with 175 *μ*L of tap water, air‐dried, and crystal violet was solubilized with 150 *μ*L of ethanol‐acetone mixture (80:20) for 30 min. The plates were scanned at 550 nm to determine the OD of the stained cells. The ODs of 3 independent plates were compared with the ODc to determine the biofilm phenotype as follows: nonbiofilm producer: OD ≤ ODc; weak biofilm producer: ODc < OD ≤ 2 × ODc; moderate biofilm producer: 2 × ODc < OD ≤ 4 × ODc; or strong biofilm producer: OD > 4 × ODc. *S*. *aureus* ATCC 33591 was used as a positive control.

### 2.6. Molecular Detection of *A*. *baumannii* Resistance Genes and *E*. *coli* Toxin Genes

Detection of *bla_OXA-51_*, *bla_OXA-23_*, and *bla_NDM-1_* genes in *A*. *baumannii* isolates was performed using primers sequences and PCR conditions as described by Turton et al. [45], Woodford et al. [46], and Wang et al. [47], respectively. *A*. *baumannii* ATCC 19606, *A*. *baumannii* ATCC BAA‐1605, and *E*. *coli* ATCC BAA‐2452 were used as positive controls in the PCR reactions, respectively. Molecular detection of the genes encoding virulence determinants in the *E*. *coli* isolates was conducted as described previously [48]. The PCR conditions were as follows: initial denaturation at 94°C for 5 min, then 30 cycles of denaturation at 94°C for 30 s, annealing at 63°C for 30 s, extension at 72°C for 1.5 min; and a final extension at 72°C for 5 min.

### 2.7. Statistical Analyses

Microbial prevalence was reported as frequencies and percentages, with 95% confidence intervals calculated for overall prevalence. Microbial counts data were log_10_‐transformed before analysis. Differences among milkshake, smoothie, and slushie samples were assessed using Pearson′s chi‐square test or the Fisher–Freeman–Halton exact test. TBC and TCC were expressed as arithmetic mean with 95% CI. Pre‐ and postenrichment counts were compared separately among beverage types using Welch′s one‐way ANOVA followed by Games–Howell post hoc tests.

The relationship between biofilm‐forming capacity and antimicrobial resistance was assessed separately within each bacterial group. Associations between biofilm phenotype and resistance burden were evaluated using Spearman′s rank correlation. Associations between individual antimicrobial resistance phenotypes and biofilm category were assessed using Fisher′s exact test. Statistical significance was defined as *p* < 0.05.

## 3. Results

### 3.1. TBC and TCC

The microbial quality of 134 beverage samples was assessed by determining the TBC and TCC. The mean counts were calculated for each beverage type both before and after an enrichment step to provide a comprehensive view of the bacterial contamination. Figure 1 shows the mean TBC and TCC across the three beverage categories. In the pre‐enrichment analysis of TBC, smoothies demonstrated the highest bacterial load (*p* < 0.05), with a mean of 3.48 × 10^8^ CFU/mL. This was greater than the mean counts observed for milkshakes (2.83 × 10^8^ CFU/mL) and slushies samples (1.72 × 10^8^ CFU/mL). The overall difference in the mean TBC among beverage categories was statistically significant (Welch′s ANOVA, *p* = 0.0015). Games–Howell analysis showed significant differences between milkshakes and smoothies (*p* = 0.00058) and between smoothies and slushies (*p* = 0.0366), whereas the difference between milkshakes and slushies was not significant (*p* = 0.443). Following the enrichment step, smoothies continued to exhibit the highest mean bacterial load (2.83 × 10^8^ CFU/mL) among the three groups. However, the mean TBC for both slushies and milkshakes decreased to 6.39 × 10^7^ CFU/mL and 2.02 × 10^8^ CFU/mL, respectively. A significant overall difference among beverage categories was detected (Welch′s ANOVA, *p* < 0.0001). Pairwise comparisons showed significant differences between milkshakes and smoothies (*p* < 0.0001) and between milkshakes and slushies (*p* = 0.0011), whereas smoothies and slushies did not differ significantly (*p* = 0.986). Consistent with the TBC findings, smoothies had the highest mean total coliform count (3.55 × 10^9^ CFU/mL), followed by slushies (1.31 × 10^8^ CFU/mL), whereas milkshakes had the lowest mean (5.84 × 10^7^ CFU/mL). The overall difference in the mean TCC among beverage types was significant (Welch′s ANOVA, *p* = 0.0301). A significant difference was identified only between milkshakes and smoothies (*p* = 0.0190). After enrichment, smoothies remained the group with the highest mean TCC (5.38 × 10^8^ CFU/mL), followed by milkshakes (4.47 × 10^8^ CFU/mL). Slushies had a lower mean TCC of 2.06 × 10^8^ CFU/mL. The overall difference among the three beverage categories was statistically significant (Welch′s ANOVA, *p* = 0.0062). Pairwise comparisons showed significant differences between milkshakes and smoothies (*p* = 0.0056) and between milkshakes and slushies (*p* = 0.0385).

**Figure 1:**
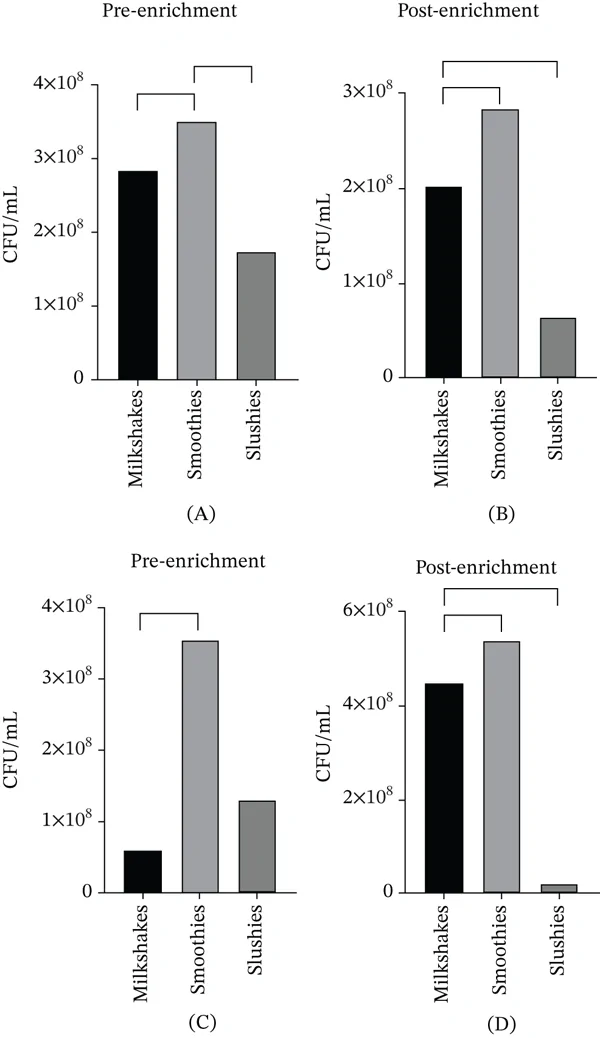
(A,B) Total bacterial count and (C,D) total coliform count for the (A,C) pre‐enrichment and (B,D) postenrichment samples. For TBC, the CFU was calculated for 39 milkshakes, 78 smoothies, and 17 slushies. For TCC, the CFU was calculated for 10 milkshakes, 52 smoothies, and 10 slushies. Bars represent arithmetic mean bacterial counts (CFU/mL). Statistical comparisons were performed using Welch′s one‐way ANOVA on log_10_‐transformed bacterial counts, followed by Games–Howell post hoc comparisons. Brackets above bars indicates significant difference. *p* < 0.05 was considered statistically significant.

### 3.2. Molecular Identification of Bacteria

A total of 171 bacterial isolates were recovered from 150 samples, including milkshakes (*n* = 41), smoothies (*n* = 87), and slushies (*n* = 22). The prevalence of the recovered bacterial groups, together with exact 95% confidence intervals and statistical comparisons among beverage types, is summarized in Table 1 and Figure 2. *Klebsiella* spp. were the most frequently isolated bacterial group, recovered from 37.3% (56/150; 95% CI: 29.6%–45.7%) of all samples. Its prevalence was significantly higher (*p* < 0.0001) in milkshakes (73.2%, 30/41) compared with smoothies (25.3%, 22/87) and slushies (18.2%, 4/22). Within the *Klebsiella* spp., *K*. *pneumoniae* was predominant, accounting for 20.7% of total isolates and exhibiting a significant concentration in milkshakes (48.8%, 20/41) relative to smoothies (11.5%, 10/87) and slushies (4.5%, 1/22) (*p* < 0.0001). In contrast, *K*. *oxytoca* exhibited low prevalence (2.7%, 4/150), with no significant variation by product type (*p* = 0.468).

**Table 1:** Prevalence of bacterial groups according to beverage type.

Bacterial group	Overall prevalence, *n* = 150, *n*/*N*(%; 95% CI)	Milkshake, *n* = 41, *n*/*N*(%)	Smoothie, *n* = 87, *n*/*N*(%)	Slushie, *n* = 22, *n*/*N*(%)	*p* value
*Acinetobacter* spp.	7/150 (4.7%; 1.9–9.4)	6/41 (14.6%)	0/87 (0%)	1/22 (4.5%)	0.0014
*Acinetobacter baumannii*	4/150 (2.7%; 0.7–6.7)	3/41 (7.3%)	0/87 (0%)	1/22 (4.5%)	0.0360
*Acinetobacter pittii*	2/150 (1.3%; 0.2–4.7)	2/41 (4.9%)	0/87 (0%)	0/22 (0%)	0.0940
*Cronobacter sakazakii*	1/150 (0.7%; 0–3.7)	1/41 (2.4%)	0/87 (0%)	0/22 (0%)	0.4200
*E*. *coli*	14/150 (9.3%; 5.2–15.2)	10/41 (24.4%)	4/87 (4.6%)	0/22 (0%)	0.0009
*Klebsiella* spp.	56/150 (37.3%; 29.6–45.6)	30/41 (73.2%)	22/87 (25.3%)	4/22 (18.2%)	< 0.0001
*Klebsiella pneumoniae*	31/150 (20.7%; 14.5–28.0)	20/41 (48.8%)	10/87 (11.5%)	1/22 (4.5%)	< 0.0001
*Klebsiella oxytoca*	4/150 (2.7%; 0.7–6.7)	0/41 (0%)	3/87 (3.4%)	1/22 (4.5%)	0.4675
*Listeria* spp.	7/150 (4.7%; 1.9–9.4)	3/41 (7.3%)	4/87 (4.6%)	0/22 (0%)	0.5626
*Listeria monocytogenes*	2/150 (1.3%; 0.2–4.7)	2/41 (4.9%)	0/87 (0%)	0/22 (0%)	0.0940
*Pseudomonas aeruginosa*	1/150 (0.7%; 0–3.7)	0/41 (0%)	1/87 (1.1%)	0/22 (0%)	1.000
*Salmonella* spp.	0/150 (0%; 0–2.4)	0/41 (0%)	0/87 (0%)	0/22 (0%)	NA
*Staphylococcus* spp.	6/150 (4.0%; 1.5–8.5)	2/41 (4.9%)	3/87 (3.4%)	1/22 (4.5%)	0.8596
*Staphylococcus aureus*	4/150 (2.7%; 0.7–6.7)	0/41 (0%)	3/87 (3.4%)	1/22 (4.5%)	0.4675
MRSA	0/150 (0%; 0–2.4)	0/41 (0%)	0/87 (0%)	0/22 (0%)	NA
*Shigella sonnei*	1/150 (0.7%; 0–3.7)	1/41 (2.4%)	0/87 (0%)	0/22 (0%)	0.4200

*Note:* Values are presented as *n*/*N* (%), where *n* is the number of positive samples, and *N* is the total number of samples tested. Exact 95% confidence intervals (CI) are provided for the overall prevalence only. Differences in prevalence among milkshakes, smoothies, and slushies were assessed using Pearson′s *χ* ^2^ test or the Fisher–Freeman–Halton exact test when expected cell counts were small. Statistical significance was defined as *p* < 0.05. NA indicates that statistical comparison was not applicable because no positive samples were detected.

**Figure 2:**
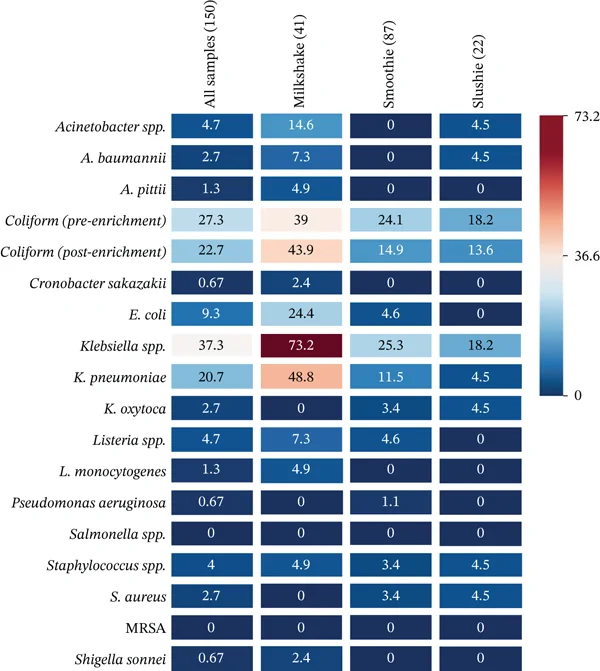
Prevalence of bacterial species isolated in this study.

*E*. *coli* was recovered from 9.3% (14/150; 95% CI: 5.2%–15.2%) of all samples, with a significant distribution across beverage types (*p* = 0.0009). Contamination with *E*. *coli* was highest in milkshakes (24.4%, 10/41), followed by smoothies (4.6%, 4/87), but was absent in slushy samples (0.0%, 0/22). Similarly, *Acinetobacter* spp. exhibited a more limited significant distribution (*p* = 0.0014), detected only in milkshakes (14.6%, 6/41) and slushies (4.5%, 1/22), but not in smoothies. *A*. *baumannii* was detected in 7.3% (3/41) of milkshakes and 4.5% (1/22) of slushies (*p* = 0.036). Postenrichment coliform prevalence was significantly elevated in milkshakes (43.9%, 18/41) compared with smoothies (14.9%, 13/87) and slushies (13.6%, 3/22) (*χ* ^2^ 15.02, *p* = 0.0005). No statistically significant differences among beverage categories were observed for *Acinetobacter pittii*, *C*. *sakazakii*, *K*. *oxytoca*, *Listeria* spp., *Listeria monocytogenes*, *P*. *aeruginosa*, *Staphylococcus* spp., *S*. *aureus*, or *Shigella sonnei* (all *p* > 0.05). DNA sequencing of presumptive isolates of *Shigella* only confirmed the identity of only one *S*. *sonnei* isolate (Table S1). *C*. *sakazakii* and *P*. *aeruginosa* were each isolated only from one sample (0.67%). No MRSA or *Salmonella* spp. isolates were recovered from any sample.

### 3.3. Antibiotic Sensitivity Testing

Antibiotic susceptibility testing of *Acinetobacter* isolates revealed high susceptibility to most agents (Figure 3). All isolates were susceptible to ampicillin/sulbactam, piperacillin/tazobactam, ceftazidime, cefepime, doripenem, meropenem, gentamicin, tobramycin, amikacin, minocycline, ciprofloxacin, and levofloxacin. Moderate intermediate susceptibility was observed for piperacillin (14.3%), ceftriaxone (42.9%), and imipenem (57.1%). In contrast, *Acinetobacter* isolates exhibited high resistance levels against tetracycline (71.4%) and doxycycline (57.1%). Trimethoprim/sulfamethoxazole showed low resistance (14.3%) and high susceptibility(85.7%). Resistance to the trimethoprim/sulfamethoxazole combination was detected in one isolate only (Figure 3).

**Figure 3:**
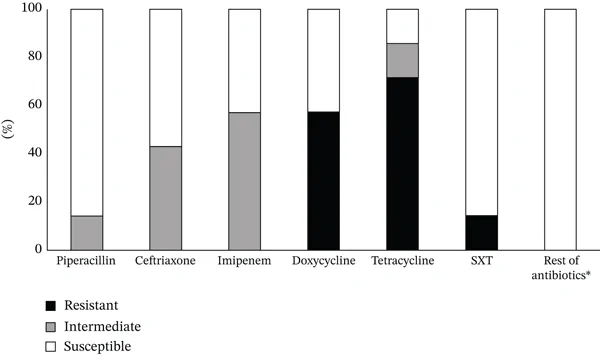
In vitro antibiotic susceptibility results for *Acinetobacter* spp. isolates (*N* = 7). The stacked bars show the percentage of isolates exhibiting resistance (black), intermediate susceptibility (grey), and susceptibility (white) to each agent tested, based on CLSI breakpoints.  ^∗^: Ampicillin/sulbactam, piperacillin/tazobactam, ceftazidime, cefepime, doripenem, meropenem, gentamicin, tobramycin, amikacin, minocycline, ciprofloxacin, levofloxacin. SXT: trimethoprim/sulfamethoxazole.

The highest resistance among the 14 *E*. *coli* isolates (Figure 4) was observed against ampicillin and sulfonamides, with 78.6% of isolates exhibiting resistance against each of these two agents. Moderate resistance rates were observed against nitrofurantoin (50%), trimethoprim (35.7%), doxycycline (35.7%), and tetracycline (35.7%). The isolates exhibited low level of resistance (14%–21%) against amoxicillin/clavulanic acid, ampicillin/sulbactam, piperacillin/tazobactam, cefotaxime, ceftriaxone, cefoxitin, ceftazidime, aztreonam, gentamicin, kanamycin, ciprofloxacin, and nalidixic acid showed low level resistance (14%–21%). All isolates were susceptible to meropenem, although 35.7% of isolates exhibited intermediate susceptibility. Similarly, high susceptibility rates were also observed for amikacin (92.9%), chloramphenicol (92.9%), aztreonam (85.7%), and gentamicin (85.7%).

**Figure 4:**
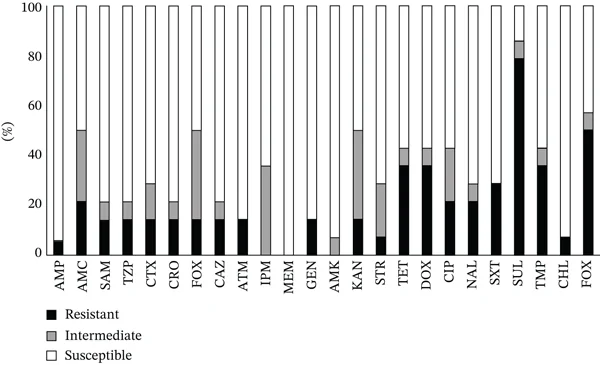
In vitro antibiotic susceptibility results for *E*. *coli* isolates (*N* = 14). The stacked bars show the percentage of isolates exhibiting resistance (black), intermediate susceptibility (grey), and susceptibility (white) to each agent tested, based on CLSI breakpoints.

All *Klebsiella* isolates were susceptible to imipenem, meropenem, doripenem, and amikacin (Table 2). In contrast, the highest resistance rate was observed against ampicillin (71.4%), followed by nitrofurantoin (33.9%). Moderate resistance rates of 16% and 21% were observed for trimethoprim/sulfamethoxazole and tetracycline, respectively. The *klebsiella* isolates exhibited high susceptibility towards piperacillin, ticarcillin/clavulanate, cefepime, cefotaxime, ceftriaxone, ceftazidime, aztreonam, gentamicin, and chloramphenicol. Intermediate susceptibility was highest for kanamycin (8.9%) and ciprofloxacin (10.7%), suggesting emerging resistance.

**Table 2:** In vitro antibiotic susceptibility results for *Klebsiella* spp. isolates (*N* = 56).

Antibiotic	Percentage of isolates		
	Resistant	Intermediate	Susceptible
Ampicillin	71.4	5.4	23.2
Nitrofurantoin	33.9	7.1	58.9
Tetracycline	21.4	5.4	73.2
Trimethoprim/sulfamethoxazole	16.1	3.6	80.4
Cefoxitin	10.7	1.8	87.5
Amoxicillin/clavulanic acid	8.9	8.9	82.1
Ciprofloxacin	3.6	10.7	85.7
Nalidixic acid	3.6	3.6	92.9
Levofloxacin	3.6	1.8	94.6
Piperacillin, ticarcillin/clavulanate, cefepime, ceftriaxone, ceftazidime, aztreonam, gentamicin, chloramphenicol	1.8	0	98.2
Cefotaxime	1.8	1.8	96.4
Kanamycin	0	8.9	91.1
Piperacillin/tazobactam, tobramycin	0	1.8	98.2
Imipenem, meropenem, doripenem, amikacin	0	0	100

*Listeria* isolates exhibited universal susceptibility against gentamicin, doxycycline, minocycline, fluoroquinolones (ciprofloxacin, levofloxacin, and moxifloxacin), trimethoprim, trimethoprim/sulfamethoxazole, rifampin, and linezolid (Figure 5). High susceptibility (85.7%) was also observed for erythromycin, tetracycline, nitrofurantoin, ceftaroline, and chloramphenicol. With respect to ceftaroline and chloramphenicol, 85.7% of *Listeria* isolates were categorized as susceptible and 14.3% as intermediate, with no resistance detected. Resistance was observed against cefoxitin (42.9%), clindamycin (28.6%), azithromycin (14.3%), erythromycin (14.3%), tetracycline (14.3%), and nitrofurantoin (14.3%).

**Figure 5:**
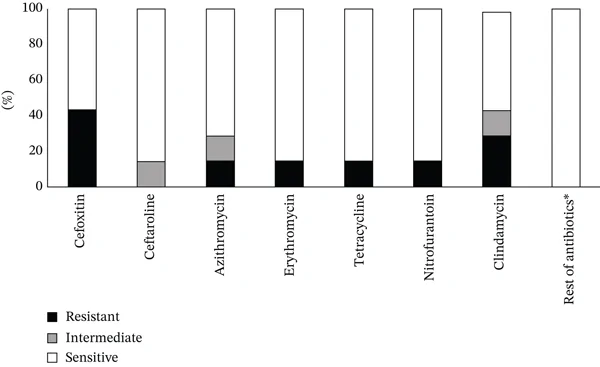
In vitro antibiotic susceptibility results for *Listeria* spp. isolates (*N* = 7). The stacked bars show the percentage of isolates exhibiting resistance (black), intermediate susceptibility (grey), and susceptibility (white) to each agent tested, based on CLSI breakpoints.  ^∗^: Gentamicin, doxycycline, minocycline, fluoroquinolones (ciprofloxacin, levofloxacin, and moxifloxacin), trimethoprim, trimethoprim/sulfamethoxazole, rifampin, and linezolid.

High antibiotic susceptibility was observed among *Staphylococcus* spp. isolates (Figure 6). All isolates were susceptible to tetracycline, doxycycline, minocycline, nitrofurantoin, chloramphenicol, and linezolid, whereas, all were resistant to azithromycin. Moreover, a high resistance rate was observed for cefoxitin (83.3%), and resistance to ciprofloxacin, levofloxacin, and trimethoprim‐related agents ranged from 33.3% to 50%. Only ciprofloxacin, moxifloxacin, and rifampin showed an intermediate resistance rate (16.7%).

**Figure 6:**
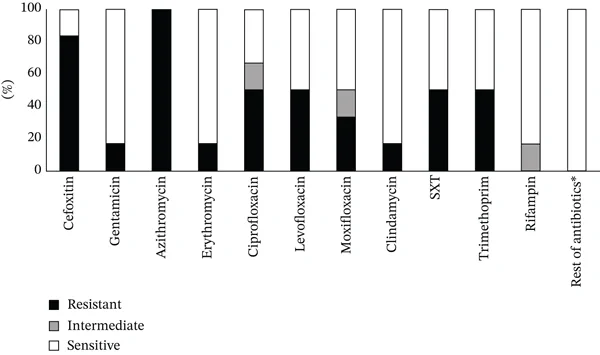
In vitro antibiotic susceptibility results for *Staphylococcus* spp. isolates (*N* = 7). The stacked bars show the percentage of isolates exhibiting resistance (black), intermediate susceptibility (grey), and susceptibility (white) to each agent tested, based on CLSI breakpoints.  ^∗^: Tetracycline, doxycycline, minocycline, nitrofurantoin, chloramphenicol, and linezolid. SXT: trimethoprim/sulfamethoxazole.

### 3.4. Biofilm Formation Assay

Biofilm‐forming capacity varied among the tested bacterial genera (Figure 7). *A*. *baumannii* showed the highest biofilm‐forming ability, with 71.4% of isolates classified as strong formers, and none categorized as nonformers. *Staphylococcus* spp. also exhibited high biofilm potential, with 66.7% of isolates classified as strong formers and only 16.7% nonformers. In contrast, *E*. *coli* showed an even distribution across all categories, with 14.3% strong, 28.6% each moderate, weak, and nonformers. *Klebsiella* spp. isolates primarily exhibited weak biofilm formation (42.9%), whereas only 10.7% were strong formers. *Listeria* spp. exhibited the lowest overall biofilm‐forming capacity, with 85.7% weak formers and no strong‐forming isolates.

**Figure 7:**
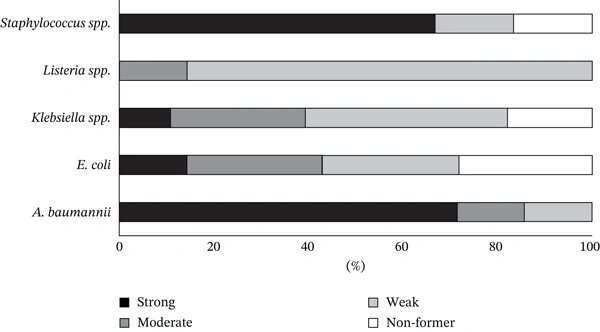
Biofilm formation phenotype for all recovered bacterial isolates in this study.

To examine whether biofilm‐forming capacity was associated with antimicrobial resistance, biofilm phenotype was correlated with the number of antimicrobial agents to which each isolate was resistant. No significant association was detected for *E*. *coli*, *Klebsiella* spp., *Acinetobacter* spp., *Listeria* spp. or *Staphylococcus* spp. In addition, no individual antibiotic exhibited a significant association with biofilm category after multiple‐testing correction. These results indicate that stronger biofilm formation was not consistently associated with a greater antimicrobial resistance burden in the isolates investigated in this study.

### 3.5. Molecular Detection of *A*. *baumannii* Resistance Genes and *E*. *coli* Toxin Genes

All *A*. *baumannii* isolates were screened for the presence of *bla_OXA-51_*, *bla_OXA-23_*, and *bla_NDM-1_* genes. All isolates tested positive for the *bla_OXA-51_* gene, whereas none f the isolated harbored the *bla_OXA-23_* and *bla_NDM-1_* genes. Moreover, *E*. *coli* isolates were screened for the presence of genes encoding virulence determinants and were negative for the following genes: *escV*, *bfpB*, *stx1*, *stx2*, *elt*, *estIa*, *estIb*, *ent*, and *invE*. On the other hand, the *aggR* gene, encoding a transcriptional regulator, was detected in 14.3% of the isolates, whereas half of the isolates tested positive for the *astA* and *pic* genes, encoding heat‐stable enterotoxin 1 and a serine protease, respectively (Table 3).

**Table 3:** Detection of virulence genes in *E*. *coli* isolates.

Isolate (source ^∗^)	Virulence gene detected
Ec14 (M)	*astA*, *aggR*, *pic*
Ec9 (M), Ec10 (M), Ec11 (Sm), Ec12 (M), Ec13 (M)	*astA*, *pic*
Ec6 (M)	*astA*
Ec3 (M)	*aggR*
Ec2 (M)	*pic*
Ec1 (M), Ec23 (Sm), Ec28 (Sm), Ec30 (Sm)	None

^∗^M, milkshake; Sm, smoothie.

## 4. Discussion

This study provides the first microbiological assessment of frozen retail beverages in Jordan, revealing that these widely consumed drinks harbor high bacterial loads, thus posing a risk to public health. Milkshakes exhibited the greatest prevalence and diversity of microbial contaminants, which were dominated by *Klebsiella* spp., *E*. *coli*, and coliforms. Smoothies demonstrated intermediate microbial contamination levels and slushies were the least contaminated. These differential contamination patterns across beverage types underscore the important interplay between ingredient composition, preparation hygiene, and the properties of the food matrix in affecting microbial safety. The predominance of coliform bacteria serves as an indicator of inadequate sanitation, whereas the detection of opportunistic pathogens such as *A*. *baumannii* and *Listeria* spp. in these products highlights a previously unrecognized food safety vulnerability. In addition, the observed trends in antimicrobial resistance and the strong biofilm‐forming capacity of some isolated species underscore the need for an urgent re‐evaluation of hygiene protocols and the implementation of targeted regulatory oversight for the frozen beverages sector.

The discrepancy in microbial contamination can be largely attributed to the compositional differences of the beverages as well as the serving temperature of each product. Milkshakes have neutral pH and are rich in dairy proteins and fats, which creates an ideal environment for bacterial proliferation, a finding consistent with previous studies identifying dairy‐based drinks as high‐risk products [1, 10]. In contrast, smoothies exhibited intermediate contamination, likely due to their lower pH and the potential presence of natural antimicrobial compounds in fruits [3, 49]. Slushies demonstrated the lowest microbial counts, likely due to their high‐sugar content and acidic composition, which is hostile to most bacterial growth.

Biofilm‐producing bacteria can persist on food‐contact surfaces and serve as sources of recurrent contamination [50, 51]. In this study, *Acinetobacter* spp. and *Staphylococcus* spp. showed the highest proportions of strong biofilm formers, whereas most *Klebsiella* and *Listeria* isolates were weak or moderate producers. Strong biofilm formers may have a greater capacity to persist on beverage‐processing equipment. However, weak biofilm formers should not be considered unimportant because biofilm formation is influenced by several environmental conditions and interactions within multispecies communities. Previous studies have similarly reported biofilm‐forming bacteria in smoothies and other food products [4, 41]. During beverage preparation, several conditions may facilitate biofilm development. Residues and moisture remaining on blenders, dispenser nozzles, tubing, and other difficult‐to‐clean components can allow bacteria to persist. In addition, repeated use may produce scratches and surface damage that provide protected sites for bacterial attachment. Several factors like surface material, organic residues, moisture, temperature, and cleaning frequency can affect biofilm development and persistence in food‐processing environments [50, 52]. These factors were not directly evaluated in the present study and therefore represent plausible explanations for the observed biofilm phenotypes rather than demonstrated causes.

A primary finding of this study is the detection of coliforms in 50% of all samples, with molecular identification confirming the presence of *Klebsiella* spp. (37.3%), *Enterobacter* spp. (18%), and *E*. *coli* (9.3%). The presence of these types of bacteria is consistent with previous reports and strongly indicates contamination from environmental sources [1, 3, 4, 10, 12, 14, 16, 17, 53–55]. The predominance of *Klebsiella* spp., and *K*. *pneumoniae* specifically, is consistent with its ecology in moist, nutrient‐rich dairy and on food‐contact surfaces with biofilms [56]. It has a very high prevalence in milkshakes (73.2%) implicates dairy ingredients (milk/ice cream) and/or inadequate sanitation of dispensing equipment [57, 58]. *Coliforms* and *E*. *coli*, which are key indicators of fecal and waterborne contamination, were frequently isolated from milkshakes, suggesting risks from water/ice, utensils, and handler hygiene [3, 58].

*Acinetobacter* spp. were isolated from the frozen drink samples at a rate of 4.7%. Most of the isolates were highly susceptible to the tested antibiotics but were strong biofilm formers. To the best of our knowledge, this is the first study to report the isolation of *Acinetobacter* spp. from milkshake samples. On the other hand, only two studies have reported the prevalence of *Acinetobacter* spp. in smoothies [4, 15]. Of the *Acinetobacter* isolates, four isolates were *A*. *baumannii*. One key factor contributing to the ability of *A*. *baumannii* to persist in various environments is its resilience on different surfaces, which may be further strengthened through interactions within polymicrobial communities [59–61]. Although the observed antibiotic susceptibility contrasts the global and local trends of MDR and extensively drug‐resistant (XDR) clinical isolates [62–64]. This finding might indicate that the *A*. *baumannii* isolates are community‐associated strains [65]. Similarly, the detection of *Listeria*, a psychrotolerant pathogen, likely resulted from using contaminated raw ingredients like unpasteurized milk or inadequately washed produce, underscoring critical failures in upstream supply chain controls [66, 67].

The antimicrobial susceptibility profiles provide a snapshot of resistance patterns circulating within the community food environment. We noted a high resistance to older, commonly used antibiotics such as ampicillin and trimethoprim/sulfamethoxazole, reflecting prolonged selective pressure. More alarming is the emerging resistance to clinically important drugs. For example, the intermediate resistance to imipenem in *A*. *baumannii* (57.1%) and *E*. *coli* (35.7%) may indicate the spread of carbapenemases. Furthermore, the high resistance of *Staphylococcus* isolates to cefoxitin (83.3%) and azithromycin (100%) is a strong indicator of community‐circulating strains. The antimicrobial susceptibility results demonstrated that most recovered isolates remained susceptible to the majority of agents tested, although resistance to selected antibiotics was observed in several bacterial groups. For example, resistance was observed to tetracycline and doxycycline among *Acinetobacter* isolates, ampicillin and sulfonamides among *E*. *coli*, and ampicillin among *Klebsiella* spp. These findings indicate the presence of specific antimicrobial resistance phenotypes in frozen beverage‐associated bacteria, but do not suggest widespread multidrug resistance.

We investigated the relationship between biofilm formation and antimicrobial resistance at the isolate level. No significant correlation was detected between biofilm‐forming capacity strength and antimicrobial resistance burden among all bacterial genera recovered. Bacteria growing within biofilms may exhibit tolerance to antimicrobial agents and disinfectants because of the protective extracellular matrix, reduced metabolic activity, and physiological heterogeneity within the biofilm. However, this biofilm tolerance is different from the acquired antimicrobial resistance measured by the conventional susceptibility testing assays. In addition, a positive relationship between the biofilm‐forming capacity and antimicrobial resistance is not universal and may vary among species and strains. An inverse relationship has recently been reported in *A*. *baumannii*, where greater biofilm‐forming capacity was associated with antimicrobial‐susceptible isolates [68]. Therefore, the strong biofilm phenotypes identified in the present study can be considered only as indicators of potential persistence rather than increased antimicrobial resistance.

## 5. Conclusion

In conclusion, this study demonstrates that frozen retail beverages in Jordan are frequently contaminated with various types of bacteria, including opportunistic species such as *K*. *pneumoniae*, *E*. *coli*, *A*. *baumannii*, *Listeria* spp., and *S*. *aureus*. Differences in bacterial prevalence and microbial loads among beverage types, together with the frequent detection of coliforms, highlight potential deficiencies in handling, equipment sanitation, and general hygienic practices during beverage preparation. Although most recovered isolates were susceptible to the antibiotics tested, resistance to selected antibiotics was observed in several bacterial groups. In addition, some isolates exhibited strong biofilm‐forming capacity, which may contribute to persistence on food‐contact surfaces. The findings of this study highlight the need for improved hygiene practices, effective cleaning and sanitation of equipment, and routine monitoring to improve the microbial quality of frozen blended beverages.

## Funding

This study was supported by the Jordan University of Science and Technology (10.13039/501100004035; 2024/508).

## Disclosure

All authors have reviewed and approved the final version of the manuscript.

## Conflicts of Interest

The authors declare no conflicts of interest.

## Supporting information


**Supporting Information** Additional supporting information can be found online in the Supporting Information section.

## Data Availability

The data that support the findings of this study are available in the Supporting Information of this article.
